# 160例老年小细胞肺癌预后多因素分析

**DOI:** 10.3779/j.issn.1009-3419.2014.01.03

**Published:** 2014-01-20

**Authors:** 筱玲 陈, 健 方, 鋆 聂, 玲 戴, 洁 张, 维亨 胡, 金娣 韩, 向娟 马, 广明 田, 森 韩, 頔 吴, 皆然 龙, 洋 王

**Affiliations:** 100142 北京，北京大学肿瘤医院暨北京市肿瘤防治研究所，恶性肿瘤发病机制及转化研究教育部重点实验室，胸部肿瘤内二病房 Key Laboratory of Carcinogenesis and Translational Research (Ministry of Education), the Second Department of Chest Cancer, Peking University Cancer Hospital and Institute, Beijing 100142, China

**Keywords:** 肺肿瘤, 老年医学, 生存期, 预后, Lung neoplsms, Geriatrics, Survival, Prognosis

## Abstract

**背景与目的:**

肺癌是目前恶性肿瘤死亡的首要原因，2/3的患者年龄超过65岁，小细胞肺癌约占全部肺癌15%-20%。本研究旨在分析65岁以上老年小细胞肺癌患者的生存状况及预后因素。

**方法:**

回顾性研究160例65岁以上老年小细胞肺癌患者临床资料，采用*Kaplan-Meier*法及*Cox*多因素回归分析预后因素。

**结果:**

① 中位随访12个月（2个月-109个月）。全组1年、3年、5年生存率分别为47.1%、13.0%、9.6%，局限期为74.4%、25.0%、19.7%，广泛期为36.8%、8.7%、5.8%。全组中位生存期（median survival time, MST）12个月，局限期24个月、广泛期11个月。全组的中位无进展生存期（progression-free survival, PFS）6个月，局限期10个月、广泛期5个月。②全组分析提示治疗前体能状态（performance status, PS）评分、治疗后PS改变、分期、有无肝转移、胸部放疗是独立预后因素。③局限期中，治疗前PS评分、胸部放疗是独立预后因素。胸部放疗方式（同步放化疗*vs*序贯放疗、早期同步放化疗*vs*晚期同步放化疗）及是否行预防性全脑放疗，MST均未见统计学差异。④广泛期中性别、治疗后PS改变、化疗方案、有无肝转移、胸部放疗、预防性全脑放疗是独立预后因素。

**结论:**

老年小细胞肺癌患者生存期与PS评分和胸部放疗相关，而广泛期患者还与性别、化疗方案、有无肝转移及是否行预防性全脑放疗相关。

## Abstract (en)

**Background and objective:**

Lung cancer is currently the leading cause of cancer death, two thirds of patients are over the age of 65. Small cell lung cancer (SCLC) accounts for about 15%-20% of all lung cancer. The objective of this study is to evaluate the survival of patients older than 65 with SCLC and analyze the independent prognostic factors in this group of patients.

**Methods:**

A retrospective study has enrolled 160 cases of lung cancer aged over 65. The prognostic factors were analyzed by *Kaplan-Meier* and *Cox* multivariate proportional hazards model.

**Results:**

① The median follow-up time was 12 (2-109) months. 1-, 3-, and 5-year survival rate was 47.1%, 13.0%, 9.6% respectively, and 74.4%, 25.0%, 19.7% for limited-stage (LD), and 36.8%, 8.7%, 5.8% for extensive-stage (ED). Median survival time (MST) of all the patients was 12 months, 24 months for LD and 11 months for ED, respectively. ② Multivariate analysis suggested that performance status (PS) pre-treatment, the change of PS after treatment, stage, liver metastases and thoracic radiotherapy were the independent prognostic factors in all patients. ③ For LD-SCLC patients, PS pre-treatment, thoracic radiotherapy were the independent prognostic factors. The model of thoracic radiotherapy (concurrent chemoradiation *vs* sequential chemoradiation, early concurrent chemoradiation *vs* late concurrent chemoradiation) and prophylactic cranial irradiation (PCI) did not show significant difference. ④ For ED-SCLC patients, sex, the change of PS after treatment, chemotherapy, liver metastases, thoracic radiotherapy, PCI were the independent prognostic factors.

**Conclusions:**

The survival time is related to PS and thoracic radiotherapy in eldly patients. Besides, it is also related to sex, chemotherapy, liver metastases and PCI for ED-SCLC.

肺癌是恶性肿瘤死亡的首要病因，美国《监测、流行病学、结局数据库》（Surveillance Epidemiology and End Results data, SEER）的数据提示68%的患者诊断时≥65岁，36.8%的患者≥75岁^[1]^，小细胞肺癌（small cell lung cancer, SCLC）占全部肺癌的15%-20%。目前SCLC的标准治疗为局限期采用放化疗结合，广泛期以化疗为主的综合治疗模式。老年患者由于合并症较多及脏器功能生理特点致其耐受性较差，化疗所致毒性增加。目前临床试验多排除老年患者，或老年患者数量极少，因此这些试验结果对老年人的适用性有限，至今尚无针对老年SCLC患者的最佳治疗方案。本研究回顾性分析160例65岁以上老年SCLC患者的生存状况，探讨影响预后的相关因素，分析各治疗方法对生存期的影响，以期对老年SCLC患者治疗提供参考依据。

## 1 资料与方法

### 1.1 临床资料

收集1994年1月-2011年12月北京大学肿瘤医院胸部肿瘤内二病房我组收治的经病理学或细胞学证实，确诊时年龄在65岁以上老年SCLC患者临床资料160例。该组患者至少接受1周期化疗并有评效结果，排除单纯手术或放疗的患者。年龄65岁-84岁、中位年龄73岁，男性120例、女性40例。采用美国退伍军人医院制定的分期标准，局限期45例（28.1%）、广泛期115例（71.9%）。70%以上伴有合并症（慢性阻塞性肺病10%、冠心病15%、脑梗塞9%、高血压33%、心律失常5%、糖尿病7.5%、消化道溃疡2.5%），28%化疗减量或延期，2.5%（4例）化疗相关死亡，2.5%（4例）因不能耐受终止治疗。

### 1.2 治疗方法

局限期主要为4个-6个周期化疗+放疗，部分患者采用手术+4个-6个周期化疗±放疗，放疗同时给予75%以上标准剂量化疗的为同步放化疗，另为序贯放疗。前3周期化疗同时接受放疗为早期同步放化疗，3周期化疗后为晚期同步放化疗。完成放化疗后缓解的患者接受预防性全脑放疗（preventive radiotherapy of whole brain, PCI）。广泛期患者以化疗为主，根据肿瘤局部退缩情况给予局部根治或姑息性放疗，化放疗后缓解患者接受PCI。化疗方案包括EP或EC方案（卡铂或顺铂+依托泊苷）126例，CAV（环磷酰胺+多柔吡星+长春新碱）8例，TAX+CBP（紫杉醇+卡铂）4例，IP（伊立替康+顺铂）10例，Topotecan+DDP（拓扑替康+顺铂）1例，CTX+DDP+VCR（环磷酰胺+顺铂+长春新碱）1例，DDP+VDS+VM26（顺铂+长春花碱+替尼泊苷）1例，GEM+CBP（吉西他滨+卡铂）1例，IFO+DDP（异环磷酰胺+顺铂）1例，TAX+EPI（紫杉醇+表柔比星）1例。

### 1.3 生存期

观察终点为死亡或末次随访，末次随访日期为2012年3月20日，总生存期（overall survival, OS）指开始治疗日期至死亡或末次随访日期，无进展生存期（progression-free survival, PFS）指开始治疗日期至肿瘤进展或死亡日期。以月为单位。

### 1.4 统计方法

所有数据录入SPSS 13.0软件数据库，单因素分析采用*Kaplan-Meier*法，生存率曲线组间比较采用*Log-rank*检验，选择单因素分析有统计学意义的变量进入多因素*Cox*比例风险回归模型分析（采用变量筛选，逐步回归法）。*P* < 0.05认为差异有统计学意义。

## 2 结果

### 2.1 随访结果

中位随访时间12个月（2个月-109个月），死亡135例（84.4%），失访2例。全组1年、3年、5年生存率分别为47.1%、13.0%、9.6%，局限期分别为74.4%、25.0%、19.7%，广泛期分别为36.8%、8.7%、5.8%。全组中位生存期（median survival time, MST）12个月，局限期24个月，广泛期11个月。全组PFS 6个月，局限期10个月，广泛期5个月。

### 2.2 预后因素分析

对患者年龄段（≥75岁与 < 75岁）、性别、治疗前体能状况（performance status, PS）、治疗后PS变化、分期、转移部位、化疗方案（EP或EC *vs*非EP或EC）、胸部放疗（局限期中同步放化疗与序贯放疗，早期同步放化疗与晚期同步放化疗）、PCI进行单因素分析。对单因素分析中具有统计意义的变量行多因素分析。

#### 2.2.1 全组

单因素分析提示疗前PS 0级-1级、疗后PS改善和稳定、局限期、无肝、肺、脑、腹腔、骨转移、胸部放疗患者生存期长（*P* < 0.05），而性别、年龄段、化疗方案不影响预后（表 1，图 1）。多因素分析显示疗前PS评分、疗后PS改变、分期、有无肝转移、胸部放疗是独立预后因素（*P* < 0.05）（表 2）。

**1:**
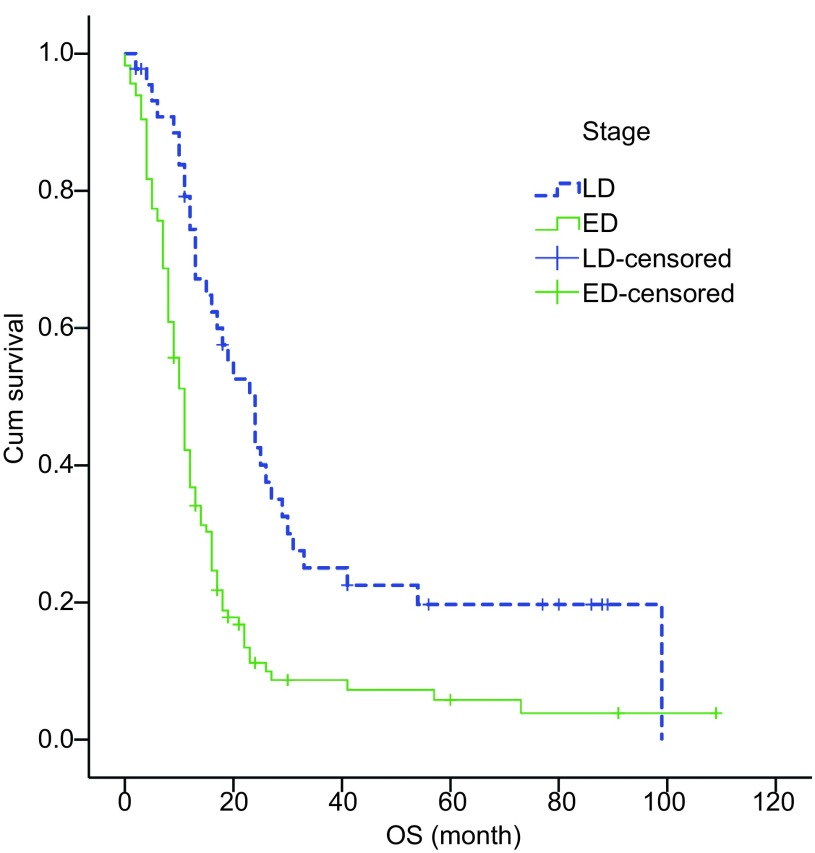
局限期和广泛期SCLC患者生存曲线（*Log-rank*检验，*P* < 0.001） Survival curves of SCLC patients with limited-stage (LD) and extensive-stage (ED) (*Log-rank* test, *P* < 0.001). OS: overall survival.

**1:** 单因素分析影响老年SCLC患者生存期的相关因素 Single analysis for survival of the total SCLC in the elderly

Factor		*n* (%)	MST (month)	95%CI (month)	*P*
Total		160	12	10.6-13.4	
Sex	Male	120 (75.0)	11	9.6-12.4	0.050
	Female	40 (25.0)	16	14.1-17.9	
Age (yr)	65-75	135 (84.4)	12	10.2-13.8	0.118
	≥75	25 (15.6)	9	4.1-13.9	
PS before treatment (ECOG)	0	14 (8.8)	23	10.1-35.9	0.007
	1	114 (71.2)	13	11.1-14.9	
	2	26 (16.2)	11	10.1-11.9	
	3	6 (3.8)	8	3.2-12.8	
The change of PS after treatment (ECOG)	Improve	71 (44.4)	13	10.1-15.9	0.001
	Stable	67 (41.9)	12	10.0-14.0	
	Worsen	22 (14.7)	4	1.3-6.7	
Stage	LD	45 (28.1)	24	18.0-30.0	< 0.001
	ED	115 (71.9)	11	9.7-12.3	
Liver	Yes	29 (18.1)	7	5.0-9.0	< 0.001
	No	131 (81.9)	14	11.6-16.4	
Lung	Yes	42 (26.3)	11	9.3-12.7	0.016
	No	118 (73.7)	13	10.1-15.9	
Brain	Yes	24 (15.0)	8	6.1-9.9	0.001
	No	136 (85.0)	13	10.8-15.2	
Abdomen	Yes	32 (20.0)	10	7.8-12.2	0.001
	No	128 (80.0)	13	10.4-15.6	
Bone	Yes	28 (17.5)	9	6.4-11.6	0.001
	No	132 (82.5)	13	9.7-16.3	
Chemotherapy scheme	EP or EC	126 (78.8)	12	10.1-13.9	0.253
	Not EP or EC	34 (21.2)	10	7.1-12.9	
Radiotherapy	Yes	79 (49.4)	17	13.7-20.3	< 0.001
	No	81 (50.6)	9	7.0-11.0	
SCLC: small cell lung cancer; PS: performance status; ECOG: Eastern Cooperative Oncology Group; EP: etoposide+cisplatin; EC: etoposide+carboplatin; MST: median survival time; LD: limited-stage; ED: extensive-stage.					

**2:** 老年小细胞肺癌*Cox*回归多因素分析 *Cox* regression of variables in the equation of SCLC in the elderly

Total				ED				LD		
Factor	*P*	RR		Factor	*P*	RR		Factor	*P*	RR
PS before treatment (ECOG)	0.024	1.396		Sex	0.017	0.563		PS before treatment (ECOG)	0.009	2.541
The change of PS after treatment	< 0.001	1.712		The change of PS after treatment	0.001	1.675		Radiotherapy	0.013	0.388
Stage	0.007	1.852		Liver	0.003	2.040				
Liver	0.003	2.028		Chemotherapy scheme	0.049	1.676				
Radiotherapy	< 0.001	0.408		Radiotherapy	0.003	0.445				
				PCI	0.015	0.256				
PCI: preventive radiotherapy of whole brain.										

#### 2.2.2 局限期

单因素分析提示疗前PS 0级，胸部放疗患者生存期长（*P* < 0.05）。同步放化疗与序贯放疗比较MST（27个月*vs* 24个月，*P*=0.813）、早期同步放化疗与晚期同步放化疗比较MST（27个月*vs* 26个月，*P*=0.270），治疗缓解患者接受PCI与未行PCI比较MST（29个月*vs* 23个月，*P*=0.288），均无统计学差异（表 3，图 2）。多因素分析示疗前PS评分和胸部放疗是局限期患者独立预后因素（*P* < 0.05）（表 2）。

**2:**
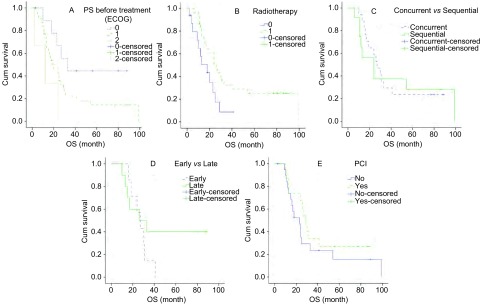
局限期SCLC患者生存曲线（*Log-rank*检验）。A：疗前PS（ECOG）0、1、2（*P*=0.045）；B：胸部放疗（Yes）与未放疗（No）（*P*=0.026)；C：胸部同步放化疗（Concurrent）与序贯放疗（Sequential）（*P*=0.813)；D：早期同步放化疗（Early）与晚期同步放化疗（Late）(*P*=0.270)；E：PCI（Yes）与未行PCI（No）(*P*=0.288)。 Survival curves of SCLC patients in LD (*Log-rank* test). A: PS before treatment (ECOG) 0, 1, 2 (*P*=0.045); B: Thoracic radiotherapy (Yes) and no radiotherapy (No)(P =0.026); C: Thoracic concurrent chemoradiation and sequential chemoradiation (*P*=0.813); D: Early concurrent chemoradiation and late concurrent chemoradiation (*P*=0.270); E: PCI (Yes) and no PCI (No) (*P*=0.288).

**3:** 单因素分析影响老年局限期SCLC患者生存期的相关因素 Single analysis for survival of the LD-SCLC in the elderly

Factor		*n* (%)	MST (month)	95%CI (month)	*P*
Total		45	24	18.0-30.0	
Sex	Male	35 (77.8)	24	18.5-29.5	0.897
	Female	10 (22.2)	16	13.1-18.9	
Age (yr)	65-75	37 (82.2)	24	14.1-33.9	0.096
	≥75	8 (17.8)	13	6.3-19.7	
PS before treatment (ECOG)	0	10 (22.2)	33	15.5-50.5	0.045
	1	32 (71.1)	20	11.9-28.1	
	2	3 (6.7)	12	0-28	
	3	0 (0)	NR	NR	
The change of PS after treatment	Improve	15 (33.3)	24	14.6-33.4	0.553
	Stable	26 (57.8)	18	8.2-27.8	
	Worsen	4 (8.9)	20	13.6-26.4	
Chemotherapy scheme	EP or EC	32 (71.1)	24	16.2-31.8	0.855
	Not EP or EC	13 (28.9)	17	7.8-26.2	
Radiotherapy	Yes	29 (64.4)	26	17.8-34.2	0.026
	No	16 (35.6)	18	7.2-28.8	
Radiotherapy pattern	Concurrent	17 (58.6)	27	18.9-35.1	0.813
	Sequential	12 (41.4)	24	7.1-40.9	
Radiotherapy time	Early	7 (41.2)	27	19.3-34.7	0.270
	Late	10 (58.8)	26	1.2-50.8	
PCI	Yes	16 (44.4)	29	24.0-34.0	0.288
	No	20 (55.6)	23	16.0-30.0	

#### 2.2.3 广泛期

单因素分析提示女性、治疗后PS改善及稳定、EP或EC方案化疗、无肝转移者生存期长（*P* < 0.05），胸部放疗者MST优于未放疗患者（14个月*vs* 10个月，*P* < 0.001），主要针对 < 2个脏器转移患者（*P* < 0.001）。治疗缓解后行PCI可延长MST（23个月*vs* 12个月，*P*=0.01）（表 4，图 3）。多因素分析示性别、治疗后PS变化、化疗方案、有无肝转移、胸部放疗、PCI是广泛期患者独立预后因素（表 2）。

**3:**
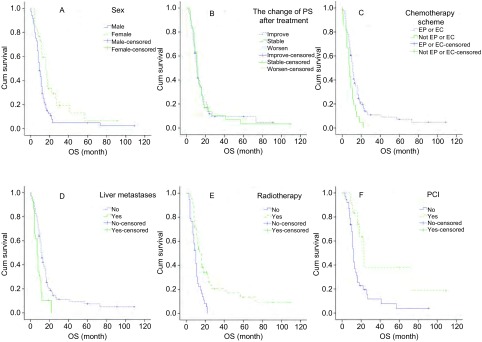
广泛期SCLC患者生存曲线（*Log-rank*检验）。A：男性和女性（*P*=0.02）；B：疗后PS改善、稳定、恶化（*P* < 0.001）；C：EP或EC方案和非EP或EC方案（*P*=0.007）；D：肝转移（Yes）和无肝转移（No）（*P*=0.001）；E：胸部放疗（Yes）与未放疗（No）（*P* < 0.001）；F：PCI（Yes）与未行PCI（No）（*P*=0.01）。 Survival curves of SCLC patients in ED (*Log-rank* test). A: Male and female (*P*=0.02); B: PS after treat improve, stable, worsen (*P* < 0.001); C: EP or EC chemotherapy scheme and not EC or EC chemotherapy scheme (*P*=0.007); D: Liver metastases (Yes) and not liver metastases (No) (*P*=0.001); E: Thoracic radiotherapy (Yes) and no radiotherapy (No) of primary site (*P* < 0.001); F: PCI (Yes) and no PCI (No) (*P*=0.01).

**4:** 单因素分析影响老年广泛期SCLC患者生存期的相关因素 Single analysis for survival of the ED-SCLC in the elderly

Factor		*n* (%)	MST (month)	95%CI (month)	*P*
Total		115	11	9.6-12.4	
Sex	Male	85 (73.9)	9	7.2-10.8	0.002
	Female	30 (26.1)	16	11.0-21.0	
Age (yr)	65-75	98 (85.2)	11	9.6-12.4	0.155
	≥75	17 (14.8)	8	5.6-10.4	
PS before treatment (ECOG)	0	4 (3.5)	10	3.1-16.9	0.523
	1	82 (71.3)	10	7.7-12.3	
	2	23 (20.2)	11	10.2-11.8	
	3	6 (5.2)	8	3.2-12.8	
The change of PS after treatment	Improve	56 (48.7)	11	9.1-12.9	＜0.001
	Stable	41 (35.7)	12	9.9-14.1	
	Worsen	18 (15.6)	4	3.0-5.0	
Chemotherapy scheme	EP or EC	94 (81.7)	11	9.6-12.4	0.007
	Not EP or EC	21 (18.3)	8	5.3-10.7	
Liver	Yes	29 (25.2)	7	5.0-9.0	0.001
	No	86 (74.8)	12	10.1-13.9	
Lung	Yes	40 (34.8)	10	8.3-11.7	0.396
	No	75 (65.2)	11	9.1-12.9	
Brain	Yes	24 (20.9)	8	6.1-9.9	0.063
	No	91 (79.1)	11	9.6-12.4	
Abdomen	Yes	32 (27.8)	10	7.8-12.2	0.073
	No	83 (72.2)	11	8.9-13.1	
Bone	Yes	28 (24.3)	9	6.4-11.6	0.087
	No	87 (75.7)	11	9.5-12.5	
Radiotherapy	Yes	50 (56.8)	14	10.7-17.3	< 0.001
	No	38 (43.2)	10	8.1-11.9	
Radiotherapy （< 2 organ metastasis）	Yes No	40 (66.7) 20 (33.3)	16 8	12.7-19.3 5.8-10.2	< 0.001
Radiotherapy (≥2 organ metastasis）	Yes No	10 (35.7) 18 (64.3)	9 11	7.5-10.5 10.1-12.0	0.891
PCI	Yes	12 (24.0)	23	16.6-29.4	0.01
	No	38 (76.0)	12	10.7-13.3	

## 3 讨论

随着期望寿命的延长及老年人肿瘤发病率的升高，肿瘤已渐成为老年人中越加普遍的疾病。肺癌是目前恶性肿瘤死亡的首要病因，2/3的患者诊断时年龄超过65岁，SCLC约占15%-20%，并且是其中恶性程度最高的一种。荷兰一项对43, 111例癌症患者的研究^[2]^发现，65岁以上癌症患者诊断时伴有一项严重合并症的为64岁以下的1.4倍，最常见的合并症为心血管疾病。由于老年人合并症多，器官功能、药物代谢和整体功能状态上也出现生理学上的变化，使治疗面临更多的挑战，年龄是否为影响老年SCLC治疗及生存的独立因素呢？本组研究中70%以上患者伴有合并症，其中严重合并症（慢性阻塞性肺病10%、冠心病15%、脑梗塞9%），28%减量或延期化疗，2.5%（4例）化疗相关死亡，2.5%（4例）不能耐受而停止治疗。本组研究结果示全组MST 12个月，局限期24个月，广泛期11个月，与文献报道的局限期MST 15个月-20个月，广泛期MST 8个月-13个月相似且局限期略优^[3]^。65岁-75岁与75岁以上年龄段比较，全组及各期均未提示年龄是其预后因素。这说明年龄是老年SCLC治疗中需要关注的问题，但不是预后指标，在临床治疗中更应关注其“功能年龄”，而非“计时年龄”。

与文献^[4]^报道相似，分期、PS评分是SCLC重要的预后因素（*P*值分别为0.007和0.024）。局限期患者生存优于广泛期，因此早期发现对延长老年SCLC患者生存至关重要。疗前PS评分是影响SCLC患者生存的独立预后因素，主要针对老年局限期患者（*P*=0.009），治疗前PS（ECOG）0级生存期长，本组局限期患者中未见ECOG 3级的患者，说明PS评分提示老年人的全身状态及治疗的耐受性，侧面反映了疾病的状态。疗后的PS变化是另一重要独立预后因素（*P* < 0.001），主要针对广泛期患者（*P*=0.001），PS改善或稳定患者预后明显好于恶化者，本组广泛期老年患者经治疗PS改善、稳定、恶化分别占48.7%、35.7%、15.6%，对于老年患者积极治疗有助于患者一般状态改善，延长生存期。

广泛期患者中性别、化疗方案、肝转移亦是独立预后因素，而脑、骨、腹腔及肺转移不影响预后。有文献^[5]^报道女性较男性预后好，本研究在广泛期中体现了这种差别（MST 16个月*vs* 9个月，RR=0.56，*P*=0.017），亦有报道^[6]^女性患者治疗中经历更多化疗相关毒性但生存期较男性患者长，不同体质及生理因素可能是其生存差异的原因，具体机制尚待进一步研究。目前EP或EC为公认的一线化疗方案，本组中EP或EC方案优于其他方案（MST 11个月*vs* 8个月，RR=1.68，*P*=0.049），因此对于老年广泛期SCLC患者该方案亦是理想的化疗方案。既往文献^[4, 7]^报道肝脏、脑或骨转移影响患者预后，本组研究显示肝脏是预后最差的转移部位（MST 12个月*vs* 7个月，RR=2.040，*P*=0.003），原因可能与肝脏本身双重血供的解剖特征相关，而脑和骨转移不影响预后，可能与本组多数脑转移患者（67%）接受全脑放疗，而承重骨转移均接受姑息放疗相关。局限期中性别，化疗方案对预后的影响未见明显差异，可能与本组老年局限期患者经综合治疗生存期长，样本量较少相关。

SCLC的标准治疗为局限期采用放化疗结合，广泛期采用化疗为主的综合治疗模式。局限期SCLC治疗中放疗是不可缺少的，本研究也显示放疗是局限期SCLC独立预后因素（*P*=0.013）。历年“NCCN指南”中局限期均推荐同步放化疗，而关于放疗方式（同步*vs*序贯，早同步*vs*晚同步）结论尚不一致，部分文献^[8]^报道早期同步放化疗是理想方案，而老年人未见相关报道。本组中同步放疗与序贯放疗相比，MST（27个月*vs* 24个月）（*P*=0.813）有延长生存趋势但无统计学差异。其可能原因是：①同步放化疗较序贯放化疗毒副反应重，老年人耐受性差，影响后续治疗；②部分患者疗程中断，或化疗剂量调整，疗效降低。近期报道的Ⅲ期临床研究^[9]^，对EP方案联合同步放疗治疗局限期SCLC放疗开始时间的比较，结果显示起始放疗组和延迟放疗组的MST分别为24.1个月和26.8个月（*P*=0.69），提示晚期同步放化疗也是合适的选择，特别是对照射野较广的患者。本组患者早期同步放化疗与晚期同步放化疗比较亦未体现差异（MST 27个月*vs* 26个月）（*P*=0.270）。胸部放疗在广泛期治疗中作用尚不明确。2012 ASCO报道了Wilson等^[10]^对胸部放疗在广泛期SCLC中的作用进行的回顾性分析，87例接受过化疗的患者中，43例接受了胸部放疗，44例患者未接受胸部放疗，MST分别为8.9个月和5.9个月（*P*=0.002）；在接受了4个-6个周期化疗的56例患者中，接受胸部放疗与未放疗患者MST分别为10.2个月和7.7个月（*P*=0.02）。本研究结果显示广泛期中胸部放疗是独立预后因素（*P*=0.003），接受胸部放疗患者预后好，MST（16个月*vs* 10个月，HR=0.445），在 < 2个脏器转移患者中体现了生存差异（*P* < 0.001），2个及以上脏器转移患者中则未见差异（*P*=0.891），提示老年广泛期患者胸部放疗可能获益，受益群体主要针对转移部位少的患者。

目前认为对局限期治疗完全缓解^[11]^和广泛期诱导化疗有效患者^[12]^进行PCI可以提高生存。本组局限期中完全及大部分缓解的患者接受PCI与未接受PCI相比（MST：29个月*vs* 23个月，*P*=0.288），有延长生存趋势但无统计学差异，考虑可能与这部分患者生存期长，例数较少相关，有待进一步研究明确。Slotman等^[12]^报道了一项随机对照研究，经4个-6个周期化疗有效的广泛期患者接受PCI可提高MST（5.4个月*vs* 6.7个月）及1年生存率（27.1% *vs* 13.3%, *P*=0.003）。本组广泛期老年SCLC治疗缓解患者接受PCI生存期延长（MST：23个月*vs* 12个月，HR=0.256，*P*=0.015），提示对广泛期治疗缓解的老年患者，PCI可能延长生存，但其对老年人神经功能损伤有待进一步评价。

综上，虽然老年患者存在较多合并症及化疗减量和延期，但并未体现年龄对生存的影响。老年SCLC局限期患者预后好，疗前一般情况好的患者生存期长，胸部放疗可延长生存；广泛期中女性、治疗后一般状态改善或稳定、无肝转移患者生存期长，EP或EC方案优于其他化疗方案，胸部放疗及对治疗缓解患者行PCI可延长生存，并且转移部位少的患者接受胸部放疗受益。胸部放疗在广泛期SCLC患者中的作用尚需前瞻性Ⅲ期随机对照研究来证实。
